# Findings from real-world clinical practice on tDCS treatment of MDD

**DOI:** 10.1192/j.eurpsy.2023.168

**Published:** 2023-07-19

**Authors:** A. Panhelainen

**Affiliations:** Sooma Oy, Helsinki, Finland

## Abstract

**Abstract:**

This study characterizes the real-world effectiveness and tolerability of transcranial direct current stimulation (tDCS) and aims to identify predictors of treatment outcome in patients with major depressive disorder (MDD). Treatment data was collected by the treating physician to a structured data collection form, from the patients who were treated with tDCS as part of routine clinical practice (F3 - F4 electrode montage, 2 mA, 30-mins sessions, 5 sessions per week, 2–3 weeks + maintenance treatment according to patient’s individual needs). Symptoms were scored according to common validated depression scales before and after the tDCS treatment. The study outcomes were clinical response (defined as >50% reduction from the baseline depression score) and remission. Furthermore, the data set allowed to investigate possible predictors of outcome, such as use of psychotropics and baseline depression severity. Overall, tDCS was found to be an effective and safe treatment for MDD in real-world patient population.

**Disclosure of Interest:**

None Declared

